# Computer Audition for Healthcare: Opportunities and Challenges

**DOI:** 10.3389/fdgth.2020.00005

**Published:** 2020-06-26

**Authors:** Kun Qian, Xiao Li, Haifeng Li, Shengchen Li, Wei Li, Zuoliang Ning, Shuai Yu, Limin Hou, Gang Tang, Jing Lu, Feng Li, Shufei Duan, Chengcheng Du, Yao Cheng, Yujun Wang, Lin Gan, Yoshiharu Yamamoto, Björn W. Schuller

**Affiliations:** ^1^Educational Physiology Laboratory, The University of Tokyo, Tokyo, Japan; ^2^Department of Neurology, Children's Hospital of Chongqing Medical University, Chongqing, China; ^3^School of Computer Science and Technology, Harbin Institute of Technology, Harbin, China; ^4^Institute of Information Photonics and Optical Communications, Beijing University of Posts and Telecommunications, Beijing, China; ^5^School of Computer Science and Technology, Fudan University, Shanghai, China; ^6^Shanghai Computer Music Association (SCMA), Shanghai, China; ^7^School of Communication and Information Engineering, Shanghai University, Shanghai, China; ^8^School of Mechanical and Electrical Engineering, Beijing University of Chemical Technology, Beijing, China; ^9^School of Life Science and Technology, University of Electronic Science and Technology of China, Chengdu, China; ^10^Department of Computer Science and Technology, Anhui University of Finance and Economics, Bengbu, China; ^11^Department of Information and Computer, Taiyuan University of Technology, Taiyuan, China; ^12^Ennova Health, Langfang, China; ^13^Department of Music Technology, Shenyang Conservatory of Music, Shenyang, China; ^14^Speech Group, AI Lab, AI Department, Xiaomi, Beijing, China; ^15^School of Precision Instrument and Opto-Electronics Engineering, Tianjin University, Tianjin, China; ^16^GLAM – Group on Language, Audio & Music, Imperial College London, London, United Kingdom; ^17^Chair of Embedded Intelligence for Health Care and Wellbeing, Augsburg University, Augsburg, Germany; ^18^audEERING GmbH, Gilching, Germany

**Keywords:** computer audition, machine learning, deep learning, artificial intelligence, health informatics, wearables, internet of things

## 1. Introduction

**Figure 1:**
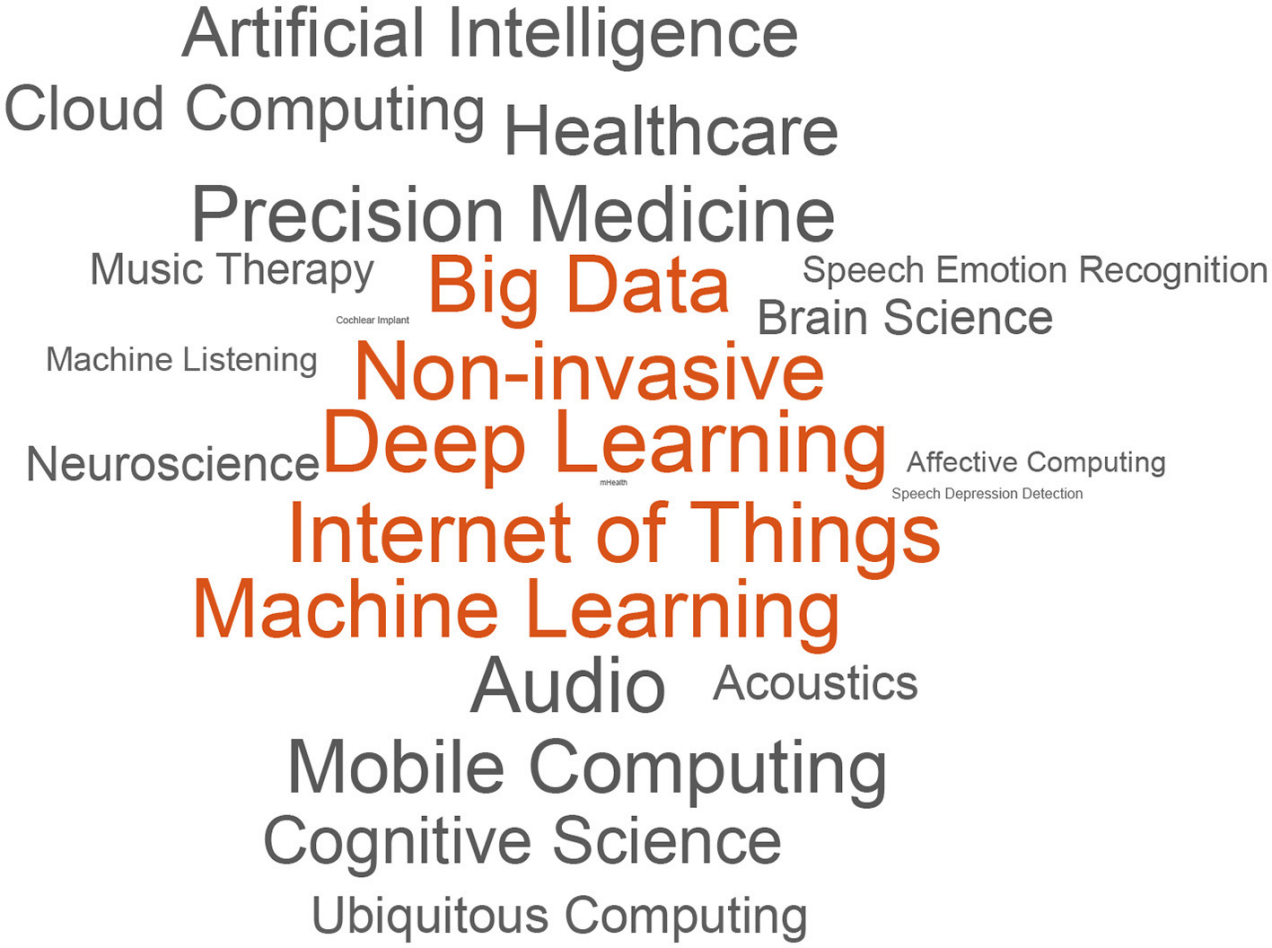
Word cloud generated by the number of references (patents and citations excluded) related to the key topics in CA for healthcare (searched by Google Scholar, years 2000 to 2019).

In the past decades, computer audition (CA), as an emerging interdisciplinary subject that includes acoustics, signal processing, machine learning, and deep learning technologies to provide computers with audio processing abilities similar to or even beyond human beings, has been increasingly studied for its applications in healthcare. Benefiting from its non-invasive characteristics, CA can facilitate both the clinical practice and home monitoring in almost every aspect of advanced intelligent medical systems like machine-listening-based diagnosis (1), mental disease screening (2), music therapy (3), and many others. On the one hand, fast development of the internet of things (IoT) and machine learning (ML) makes it easy to collect and analyze the health-related audio data using the most prevalent devices. On the other hand, even though the market/demand is great, CA for healthcare applications is still a young field compared to automatic speech recognition (ASR) (4) and music information retrieval (MIR) (5). To provide an overview of CA for healthcare concerns, Figure 1 shows a word cloud generated by key topics related to CA for healthcare in the past two decades on Google Scholar.

A forum on future audio technologies for healthcare was organized at the Harbin Institute of Technology, which was held on 28 December 2019 during the 7th Conference on Sound and Music Technology (CSMT) in Harbin, P. R. China^1^. This forum and its summary report in this paper present the current consensus and opinions from a broad range of leading scientists from the expertise of audio technologies, mobile Health (mHealth), IoT, AI, smart wearables, cognitive sciences, neuro sciences, biomedical engineering, and clinical practice. The authors hope this discussion can be a good start for not only attracting more attentions for this promising interdisciplinary field but also for providing a venue for colleagues from multiple fields to understand where we are and the future trends in the development of CA for healthcare.

## 2. Clinical Demands and Big Data

In clinical practice, demand is increasing for personalized and human-centered medical service. The cutting-edge technologies in ML and its subset, *deep learning* (DL) (6), are increasing the capabilities of CA to play an important role in medical applications. Moreover, it is even easier to capture big audio data from the increasingly prevalent sensor devices now used in daily life. The question of how to leverage the power of AI and Big Data to better the healthcare field is now attracting attention and global efforts.

## 3. Non-invasive mHealth Applications

Audio-based methods are marked by being cheap, convenient, and—most importantly—non-invasive. Whether analyzing the audio signals generated by the human body [e.g., snore sounds (1)], or using music for treatment of mental diseases (3), subjects have no need to be equipped with multiple sensors or even be burdened by invasive devices (e.g., endoscopy). Additionally, CA can make it feasible to collect data from subjects via mobile devices (e.g., a smartphone), which can provide the subjects 24 × 7 monitoring service.

## 4. Data Collection, Annotation, and Partition

Open access databases are crucial for a sustainable and reproducible research. However, CA for healthcare is lacking in standard available public databases. Numerous works were presented by using private databases, which limits the comparability and objectivity of studies on algorithms and methods. In the future, collecting and releasing more publicly accessible databases needs to be a top priority. For a specific topic, the data acquiring system (equipment, environment, place) should be consistent, aiming to minimize the effects caused by humans. Unlike normal applications in other CA fields of application (e.g., speech recognition), healthcare-related projects need specific domain knowledge (e.g., medicine). Annotation of databases is another tough mission. On the one hand, there is a large amount of unlabeled data that can be easily collected by ubiquitous microphone devices. On the other hand, accurately labeled, clean, and high-quality data are rare. To address this (labeled) *data scarcity* issue, unsupervised learning (7), semi-supervised learning (8), active learning (9), and synthesis, such as by generative adversarial networks (GANs) (10), can be explored more in the healthcare area.

## 5. Evaluation Metrics

Using suitable and reasonable evaluation metrics is necessary to guarantee a high-quality control progress in developing algorithms and methods. In healthcare applications, screening (such as binary classification of *normal* or *abnormal*) is the prerequisite for almost all cases. Thus, the widely used evaluation metrics in existing works are *accuracy, sensitivity, specificity*, and *precision*. However, data imbalance is a prevalent phenomenon in numerous healthcare applications. Moreover, multi-class classification and regression can be more accurate than only screening in clinical practice. Hence, *unweighted average recall* (UAR) (11) is advised rather than the frequently used accuracy due to the latter potentially leading to over-optimistic conclusions. In addition, confusion matrices, the receiver operating characteristic curve, and derived measures such as the area under curve or equal error rate, can provide better insight into a model's performance.

## 6. Fundamental Research

Fundamental research is always crucial and beneficial for CA applications in healthcare. In a classic ML paradigm, human hand-crafted features can be good indicators for researchers to know the relationship between the acoustical properties (in time and frequency domain) and the pathological symptoms. For instance, the popular large-scale acoustic feature extraction toolkit openSMILE (12) can provide thousands of well-designed features that can be used both for the statistical analysis and the ML model building. DL, as a hot sub-discipline of ML, is currently dominating most of the works in AI applications due to its powerful capacity to learn higher representations directly from big data. Particularly, deep end-to-end system can learn features themselves from raw audio data without any human domain knowledge (13). Nevertheless, it is difficult to build an explainable and responsible AI system by DL. In particular, finding the underlying mechanism of the pathological symptoms can never be neglected in any medicine-related subject. We believe that future work should be done by combing both the classic ML and DL methods. Understanding the fundamental knowledge is equally important for building strong enough models.

## 7. Efficient Collaboration Across Multiple Fields

As indicated in (14), collaborations across fields of expertise can benefit both the computational scientists and the experimentalists for ML applied to life sciences and medicine. However, breaking the walls between each subject (e.g., medicine and engineering) is still something that needs doing. Experts from an engineering background may look more into the state-of-the-art technologies that can be used but pay less attention to the real clinical practice or subjects' requirement. Medical scientists usually have a stronger interest to uncover the pathology via the help of AI but show less passion for the mechanisms of ML methodologies. However, in order to achieve a major breakthrough, an efficient and thorough collaboration between all involved experts is a prerequisite. Specifically, CA for healthcare needs even more fields involved, e.g., arts, education, and ethics.

## 8. Intellectual Property Protection

The intellectual property (IP) protection is always essential for high-tech research and development. In particular, due to the interdisciplinary characteristic, IP protection cannot be well-implemented by a single field. On the side of experts from a medical background, the data itself should be fully considered as their IP. However, it should be encouraged to publicly release the data for scientific purposes. On the engineering end, the efforts toward developing algorithms, platforms, software, etc., should be valued.

## 9. Discussion

We fully consider the aspects of clinical demands and big data, non-invasive mHealth applications, data collection, evaluation metrics, fundamental research, efficient interdisciplinary collaboration, and IP protection. We believe that, by reading this brief opinion piece, readers can gain a clear insight on where we are and what we can do in the future by CA for healthcare in this often under catered for area of artificial intelligence (AI). In summary, CA for healthcare is a young and promising field that needs tremendous collaboration across different fields. Future work should aim at the development of non-invasive clinical apparatus, in-home health monitoring system, and personal precision treatment service.

## Author Contributions

All the co-authors contributed to this work. KQ chaired the forum during CSMT 2019 and organized the writing work of this paper. KQ, YY, and BS conducted this summary and co-wrote this paper. XL, HL, SL, WL, ZN, SY, LH, GT, JL, FL, SD, CD, YC, YW, and LG actively discussed and participated in this work.

## Conflict of Interest

CD was employed by the company Ennova Health. YW was employed by the company Xiaomi. BS was employed by the company audEERING GmbH. The remaining authors declare that the research was conducted in the absence of any commercial or financial relationships that could be construed as a potential conflict of interest.

## Footnotes

^1^ http://www.csmcw-csmt.cn/
